# *Cytauxzoon conepatus* n. sp. (Piroplasmida: Theileriidae) infecting *Conepatus semistriatus* (Mephitidae) in Brazil

**DOI:** 10.1016/j.ijppaw.2026.101297

**Published:** 2026-10-03

**Authors:** George Magno Sousa do Rêgo, Ana Paula Nunes de Quadros, Matheus Almeida Duarte, Liliane Maria do Rosario Batista, Paula Damasceno Gomes, Giane Regina Paludo

**Affiliations:** a Laboratório de Patologia Clínica Veterinária, Faculdade de Agronomia e Veterinária, FAV, Universidade de Brasília, UNB, Distrito Federal, Brasília, Brazil; b Jaguaracambé, Associação para Conservação da Biodiversidade, Distrito Federal, Brasília, Brazil; c Centro de Conservação e Educação Socioambiental Parque Vida Cerrado, Barreiras, Bahia, Brazil; d Departamento de Clínica e Cirurgia Veterinárias, Escola de Veterinária, Universidade Federal de Minas Gerais, Belo Horizonte, Minas Gerais, Brazil

**Keywords:** Carnivora, Apicomplexa, Conepatus, PCR, Piroplasmida

## Abstract

The genus *Cytauxzoon* comprises apicomplexan hemoparasites primarily associated with domestic and wild felids, with occasional reports in non-felid carnivores. This study reports the first detection and molecular characterization of *Cytauxzoon conepatus* n. sp. infecting *Conepatus semistriatus* (Mephitidae), thereby expanding the known host range of the genus in South America. Two free-ranging, clinically healthy individuals were captured in the Cerrado biome of western Bahia, Brazil. Intraerythrocytic piroplasm forms consistent with *Cytauxzoon* spp. were observed in blood smears, and infection was confirmed by conventional PCR. Phylogenetic analyses based on nuclear (18S rRNA) and mitochondrial (*cytochrome b* and *cytochrome c* oxidase subunit I) genes consistently revealed a distinct and well-supported monophyletic species, clearly separated from *Cytauxzoon brasiliensis*, *C. felis*, and other described species. The 18S rRNA sequences showed high nucleotide identity with American *Cytauxzoon* species (99.3-99.4%) and low p-distance values (0.006-0.007), whereas comparisons with European and Asian species yielded substantially higher p-distances (≥0.030). Mitochondrial markers exhibited greater genetic divergence: *cytb* p-distance values ranged from 0.074 to 0.077 in comparisons with *C. felis* and from 0.082 to 0.084 with *C. brasiliensis*, while *cox-1* analyses revealed p-distance values of approximately 0.087-0.091 relative to both species, with nucleotide identity consistently below 92%. The congruent signal obtained from nuclear and mitochondrial markers supports the recognition of *Cytauxzoon conepatus* n. sp. as an evolutionarily distinct species, potentially endemic to the Brazilian Cerrado. These findings suggest that *Cytauxzoon* diversity in Neotropical ecosystems has been substantially underestimated and emphasize the importance of expanding multilocus molecular surveys to include non-felid carnivores.

## Highlights

• *Cytauxzoon conepatus* n. sp. was identified in *Conepatus semistriatus* in Brazil.
• Multilocus analyses supported a distinct monophyletic *Cytauxzoon* species.
• Mitochondrial markers supported differentiation from related *Cytauxzoon* species.
• Infected *C. semistriatus* were clinically healthy, suggesting a subclinical infection.
• Non-felid carnivores may harbor underestimated *Cytauxzoon* diversity.

## Graphical abstract

**Figure:**
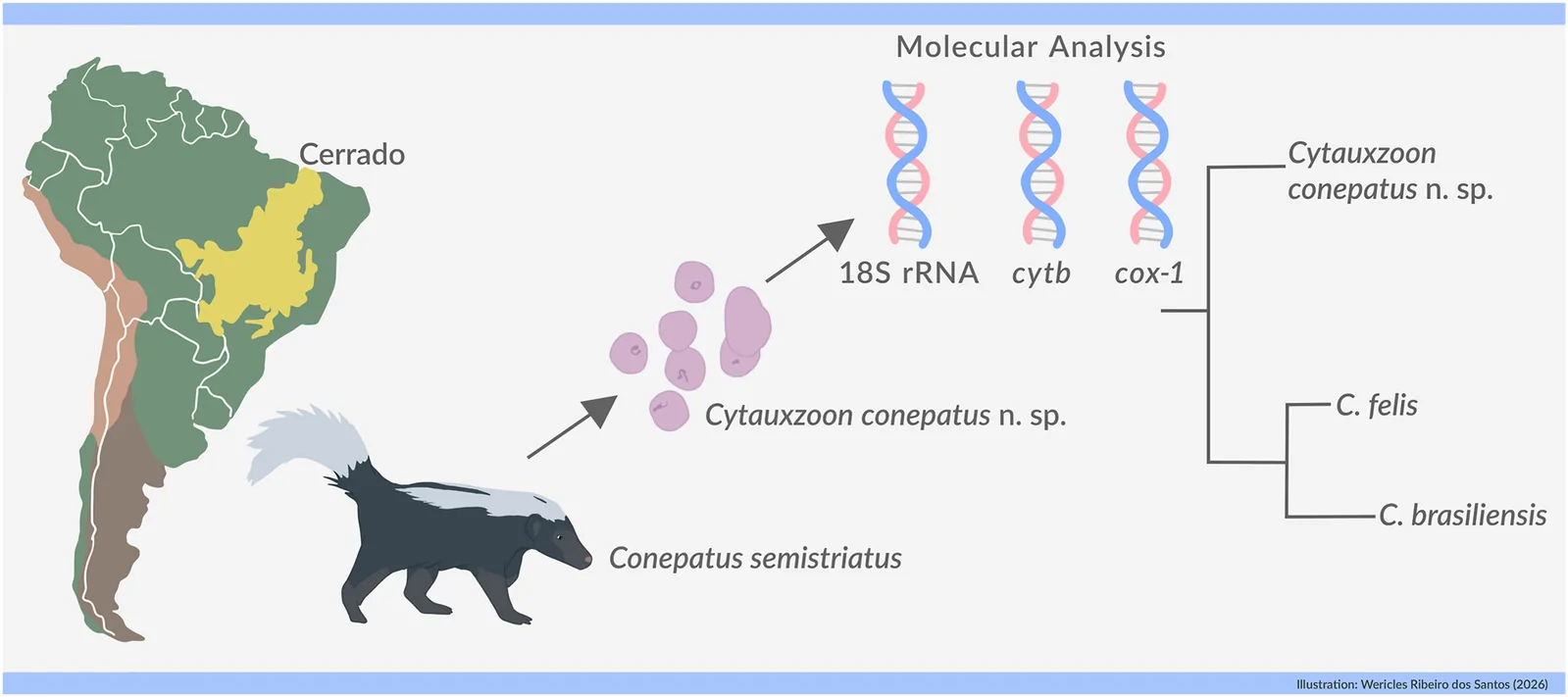

## 1 Introduction

*Cytauxzoon* spp. is an apicomplexan protozoan that was first described in the late 1940's, infecting a deer *(Sylvicapra grimmia)* in sub-Saharan Africa (Neitz and Thomas, 1948). Nowadays, this apicomplexan agent primarily infects domestic and wild felines (Alves et al., 2025; Calchi et al., 2024; Willi et al., 2022). However, other species within the Order Carnivora, such as meerkats (*Suricata suricatta*) (Leclaire et al., 2015), Hokkaido brown bear (*Ursus actos yesoensis*), japanese brow bear (*Ursus thibetanus japonicus*) (Moustafa et al., 2020), civet (*Paradoxurus musangus*) (Chong et al., 2025) and recently hyenas (*Hyaena hyaena*) (Sazmand et al., 2026) have already been recorded as hosts.

To date, seven species of the genus *Cytauxzoon* have been described, namely: *Cytauxzoon felis* (Wagner, 1976), described in the United States; *Cytauxzoon banethi, C. otrantorum, C. europaeus* (Panait et al., 2021)*,* described in Europe; *C. brasiliensis,* described in Brazil (Duarte et al., 2024) *Cytauxzoon manul* (Reichard et al., 2005) and *Cytauxzoon paradoxurus* (Chong et al., 2025) described in Asia. In this taxonomic picture, in addition to the recently described *C. brasiliensis*, other distinct *C. felis* genovariants are reported in Brazil (Calchi et al., 2025a, 2025b).

Transmission of species of the genus *Cytauxzoon* occurs through the inoculation of sporozoites during the blood meal of infected ixodid ticks. *Amblyomma americanum* and *Dermacentor variabilis* are the confirmed vectors for *C. felis* in the United States (Allen et al., 2019; Blouin et al., 1984; Reichard et al., 2009). In Brazil, *Amblyomma sculptum* has been proposed as a putative vector of *Cytauxzoon* spp. based on both epidemiological (Fagundes-Moreira et al., 2022) and molecular evidence (Santana et al., 2022). Recently, the detection of *Cytauxzoon* spp. DNA in *Ixodes ricinus* collected from the environment in Europe has been reported (Meli et al., 2025). For the other described species of the genus, it is presumed that transmission also occurs through ixodid ticks, although the specific vectors have not yet been determined, and there is no experimental evidence to date that elucidates this vectorial relationship.

Infection caused by *C. felis* is a potentially lethal disease in domestic cats (*Felis catus*), and can rapidly progress to severe clinical conditions, especially in its acute form. However, subclinical infections are also described, in which animals remain asymptomatic, acting as reservoirs of infection for new individuals (Reichard et al., 2009; Sherrill and Cohn, 2015; Wagner, 1976; Zieman et al., 2023). In wild felids, the infection is usually subclinical, making them, like domestic cats, natural reservoirs of the hemoparasite and playing a crucial role in its persistence and spread in the wild (Alves et al., 2025; Obiegala et al., 2024; Reichard et al., 2010). Nevertheless, clinical and fatal cases of *C. felis* infection have also been reported in wild felids, particularly in captive environments (Guizelini et al., 2021; Peixoto et al., 2007).

Diagnosis of *Cytauxzoon* spp. infection can be made by direct observation of intra-erythrocyte merozoites and trophozoites in blood smears or schizonts in cytological or histopathological preparations of tissues such as liver, spleen, and lymph nodes (Duarte et al., 2024; Reichard et al., 2024; Sleznikow et al., 2022). Furthermore, polymerase chain reaction (PCR), using different genes, can also be employed in diagnosis because it is a sensitive and highly specific diagnostic method, especially in infections with low parasitemia (Birkenheuer et al., 2003; Kastl and Springer, 2023).

The application of molecular tools has made it possible to expand knowledge about the described species of the genus, including descriptions of new species such as *C. banethi, C. otrantorum, C. manul* (Reichard et al., 2005; Panait et al., 2021), *C. brasiliensis* (Duarte et al., 2024) and *C. paradoxurus* (Chong et al., 2025). Despite these advances, *Cytauxzoon* infections remain an area to be explored, particularly in the Brazilian Cerrado, whose faunal richness contrasts sharply with the scarcity of research focused on the detection, diversity, and clinical-pathological impact of these protozoa in wild carnivores.

Although knowledge on the subject has evolved, there is still a significant gap regarding the species diversity and geographic distribution of *Cytauxzoon* spp. in non-feline hosts. This scarcity of information hinders the understanding of the parasite's ecology and its risks to the conservation of host species in the world's most biodiverse savanna (Strassburg et al., 2017). In light of this, the present study aimed to describe the occurrence of *Cytauxzoon* in *Conepatus semistriatus* (Mephitidae) from the Cerrado region of western Bahia and to characterize the species found through molecular analysis.

## 2 Materials and methods

### 2.1 Ethical aspects

All procedures performed were approved by the Animal Use Ethics Committee (CEUA) of the University of Brasília, registered under protocol number 23106.125953/2020-59, and registered in SISBIO (Biodiversity Authorization and Information System) under number 85850-1.

### 2.2 Capture and sampling

Two specimens of *Conepatus semistriatus* (Boddaert, 1785) were sampled during a free-ranging carnivore capture campaign conducted in July 2022 on rural properties located between the municipalities of Barreiras and Luís Eduardo Magalhães, in the far western mesoregion of Bahia (11.8063° S, 46.0361° W).

Live-box traps constructed of iron and NR12 galvanized steel with a 3 × 3 cm mesh were used for capture. To increase capture success during the campaigns, an acclimatization period was implemented, during which the traps remained open and baited for at least seven days before the capture began. After the capture began, which lasted 10 consecutive days, the traps remained open 24 h a day and were checked twice a day, at 7:00 a.m. and 4:00 p.m., to minimize stress on the captured animals. The baits used were cooked chicken, sardines, bacon and seasonal fruits, which were replaced every two days, and the points were monitored by camera traps.

Once captured, the animals were subjected to chemical restraint using a combination of tiletamine hydrochloride and zolazepam (Zoletil, Virbac, Carros, France) at a dose of 4 mg/kg (IM) based on their visually estimated weight. After loss of consciousness, the animals were removed from the traps and weighed. Anesthesia was monitored throughout the procedure by measuring physiological parameters such as heart rate, respiratory rate, and rectal temperature.

Blood collection was performed by venipuncture of the jugular vein into tubes containing anticoagulant (EDTAK^3^) and into tubes containing clot activator to obtain serum. The volume of blood collected did not exceed 1% of the animal's body weight. Blood samples were kept refrigerated between 4 and 8°C until initial processing (hematological analysis). Whole blood and blood serum samples were frozen at −20°C for subsequent DNA extraction and biochemical tests, respectively, performed at the Veterinary Clinical Pathology Laboratory of the Faculty of Veterinary Medicine (FAV) of the University of Brasília (UnB).

To ensure individual identification of captured *C. semistriatus* specimens, temporary marking was performed by clipping the hairs on the distal end of the tail. This procedure is based on techniques previously validated for similar-sized carnivores, such as *Procyon lotor,* in which clipping the caudal hairs has proven to be an efficient, quick-to-perform, minimally invasive method suitable for short-term studies (Geller, 2020).

### 2.3 Hematological and biochemical analysis

EDTA blood samples were processed and analyzed in a field laboratory located near the capture regions. Blood cell counts were performed manually using a hemocytometer chamber, as described by (Macneill, 2015). Packed cell volume (%) was determined by centrifuging a capillary filled with whole blood at 10,000 rpm for 5 min and then reading it on a specific table (Goldenfarb et al., 1971). A BIO 2000 semi-automatic spectrophotometer (Bioplus, Barueri, SP, Brazil) was used to determine hemoglobin concentration (g/dL).

Once the blood test results were obtained, erythrocytes indices (MCV: mean corpuscular volume; MCHC: mean corpuscular hemoglobin concentration) were obtained using standard calculations. Blood smears were prepared and stained with diff-quick stain (Laborclin, Pinhais, PR, Brazil) to perform the differential leukocyte count and manual platelet estimation. The differential leukocyte count was performed by counting 100 leukocytes using the immersion objective (x100). The platelet estimate was calculated from the number of platelets visualized in 10 fields using the immersion objective and using the following formula: x=2.000.y, where *y* is the number obtained from counting the platelets contained in the 10 immersion fields (Nosanchuk et al., 1978; Weiser, 2012).

Samples collected in tubes containing clot activator were centrifuged at 2500 rpm for 10 min to obtain serum and stored in microtubes. The serum was frozen at −20°C and used to determine serum enzymatic activity of alanine aminotransferase (ALT), aspartate aminotransferase (AST), alkaline phosphatase (ALP), and quantification of urea, creatinine, total protein, and albumin using a Cobas C-111 automated analyzer (Roche Diagnostics, Rotkreuz, Switzerkand). Globulins were obtained by calculating the difference in concentrations of total protein and albumin.

### 2.4 Parasitological and molecular assays

The direct detection of the hemoparasites was conducted by blood smears evaluation under the light microscope using 40X and 100X objectives, according to the methodology described by De Oliveira et al. (2022).

Conventional polymerase chain reactions (cPCR) were carried out at the Veterinary Clinical Pathology Laboratory, College of Veterinary Medicine (FAV) at the University of Brasília (UnB). DNA extraction was performed from blood collected in EDTA using the Illustra Blood Genomicprep Kit (GE Health Care, Little Chalfont, Buckinghamshire, UK) according to the manufacturer's instructions. To assess the viability of the extracted DNA and validate the extraction of all samples, cPCR was performed to confirm the presence of the gene encoding the enzyme glyceraldehyde-3-phosphate dehydrogenase (*gapdh*) (Birkenheuer et al., 2003).

After confirming DNA viability using the *gapdh* gene, the samples were subjected to cPCR screening for agents of the Piroplasmida order, using a 409 base pair (bp) fragment of the 18S rRNA gene as a target (Carret et al., 1999). Positive samples in the screening were subjected to additional cPCRs (18S rDNA, cytochrome b - *cytb* and cytochrome c oxidase subunit I - *cox-1* genes) to characterize the agent involved according to protocols described by Panait et al. (2021) and Corduneanu et al. (2017). All samples were tested in duplicate and a sample known to be positive for *C. brasiliensis* (PP583821) (Duarte et al., 2024) was used as a positive control and Mili-Q water was used as a negative control.

The amplification products of the cPCR reactions were separated by 1% agarose gel electrophoresis and visualized after staining with ethidium bromide (Vetec Sigma-Aldrich, St Louis, MO) using an ultraviolet transilluminator (UV transilluminator, UVP LLC, Upland, CA). The cPCR products of the 18S rRNA, *cytb* and *cox-1* genes were purified using a commercial kit PureLink Quick Gel Extraction & PCR Purification Combo Kit (Invitrogen, Calsbad, CA) following the manufacturer's recommendations. After purification, the samples were sequenced by the Sanger method at the Center for Biological Resources and Genomic Biology (CREBIO) of UNESP Jaboticabal, São Paulo.

### 2.5 Molecular cloning

The 18S rDNA cPCR products larger than 1500 bp were subjected to cloning. The pGEM-T Easy vector (Promega, Madison, WI, USA) was used for cloning according to the manufacturer's instructions. Using the Blue/White colony system, one clone from each sample was selected, and after confirming the presence of the gene of interest by cPCR, plasmid DNA extraction/purification was performed using the Wizard Plus SV Miniprep DNA Purification System (Promega, Madison, WI, USA). After purification, the plasmid DNA was subjected to Sanger sequencing at the Center for Biological Resources and Genomic Biology (CREBIO) of UNESP Jaboticabal, São Paulo with the following oligonucleotides: M13F (5′CGCCAGGGTTTTCCCAGTCACGAC-3′) / M13R (5′GTCATAGCTGTTTCCTGTGTGA-3′) (Lau et al., 2013).

### 2.6 Phylogenetic analysis

Maximum-likelihood phylogenetic trees were performed to investigate the evolutionary relationships of the *Cytauxzoon* sequences identified in the present study, using three distinct molecular markers: partial 18S rRNA, *cytb* and *cox-1* genes. The dataset comprised 155 sequences of 18S rRNA (1056 bp), 124 sequences of *cytb* (622 bp), and 98 sequences of *cox-1* (552 bp). For each gene, multiple alignments were generated using the ClustalW algorithm. All available *Cytauxzoon* sequences from the GenBank corresponding to the same gene regions were included. Phylogenetic trees were reconstructed in RAxML-NG v1.2.1. *Babesia vogeli* (AY072925) *Hepatozoon* sp. (MN245143), and *Theileria parva* (MW172709) were used as outgroups for the 18S rRNA, cytb, and *cox-1* datasets, respectively. Model selection was carried out using jModelTest v2.1.10, which identified HKY + G + I as the most suitable nucleotide substitution model for both the 18S rRNA and *cytb* alignments, whereas HKY + G was determined as the best model for the *cox-1*. Branch support was assessed with 1000 bootstrap replicates, and convergence was verified in all analyses.

An additional maximum-likelihood phylogenetic analysis based on the partial 18S rRNA gene was performed to assess the placement of the *Cytauxzoon* sequences identified in the present study within the broader phylogenetic framework of the order Piroplasmida. The dataset comprised 94 sequences (1012 bp), including representative sequences of the Piroplasmida groups available in GenBank for the corresponding gene region. Multiple sequence alignment was performed using the ClustalW algorithm, and the phylogenetic tree was reconstructed using RAxML-NG v1.2.1. *Cardiosporidium cionae* (EU052685) was used as the outgroup. The HKY + G + I nucleotide substitution model was selected using jModelTest v2.1.10. Branch support was assessed using 1000 bootstrap replicates, and convergence was verified. Details of the sequences included in all phylogenetic analyses are provided in the Supplementary Material1.

## 3 Results

The two sampled individuals of the species *Conepatus semistriatus*, both males, hereinafter identified as ID 1 and ID 2, were captured on the same property and at the same sampling point (11.8063° S, 46.0361° W) on different days. The hematological and biochemical values obtained from the blood samples of the captured individuals are presented in Table 1, Table 2, respectively. In the blood films prepared from the analyzed samples, intraerythrocytic structures with a signet ring shape (Fig. 1) were visualized in both individuals, compatible with *Cytauxzoon* sp.

**Table 1:** Hematological values of two individuals of striped hog-nosed skunk (*Conepatus semistriatus*) sampled in western Bahia.

	ID 1	ID 2	Reference interval *(Mephitis mephitis)* (Miller, 2019)
**Erythrogram**			
PCV(%)	36	36	24.0 - 53.9
RBC(x10^6^/μL)	5.6	6.2	5.28 - 11.13
Hemoglobin (g/dL)	12.6	12.2	7.5 - 17.1
MCV (fL)	64.2	58.1	39.3 - 60.5
MCHC (g/dL)	35.0	33.9	27.3 - 38.1

**Leukogram**			
WBC (x10^3^/μL)	6.9	9.6	1.68 - 11.96
Neutrophils (/uL)	5865	6144	510 - 7050
Lymphocytes (/uL)	750	3264	620 – 5550
Monocytes (/uL)	138	192	36 - 788
Eosinophils (/uL)	138	0	31 – 1238
Basophils (/uL)	0	0	-

**Platelets indices**			
Platelets (/uL)	112,000	186,000	37,000–575,000

**Others**			
Total plasma protein (g/dL)	8.8	8.2	-

ID1/ID2: captured specimens of *C. semistriatus*; PCV packed cell volume; RBC: red blood cell; MCV: mean corpuscular volume; MCHC: mean cell hemoglobin concentration; WBC: white blood cell.

**Table 2:** ***-*** Biochemical values of two individuals of striped hog-nosed skunk (*Conepatus semistriatus*) sampled in western Bahia.

	ID 1	ID 2	Reference interval (Miller, 2019)
Urea (mg/dL)	**78**	**92**	10 - 42
Creatinine (mg/dL)	0.6	0.5	0.2 - 1.0
ALT (UI/L)	54	195	29 - 323
AST (UI/L)	**24**	**42**	47 - 171
Alkaline phosphatase ALP (UI/L)	11	75	5 - 147
Total protein (g/dL)	7.0	6.8	4.8 - 8.9
Albumin (g/dL)	3.2	3.9	2.1 - 4.3
Globulin (g/dL)	3.8	2.9	2.0 - 6.0

ID1/ID2: captured specimens of *C. semistriatu*s.

Values outside the reference interval are shown in bold.

**Fig. 1:**
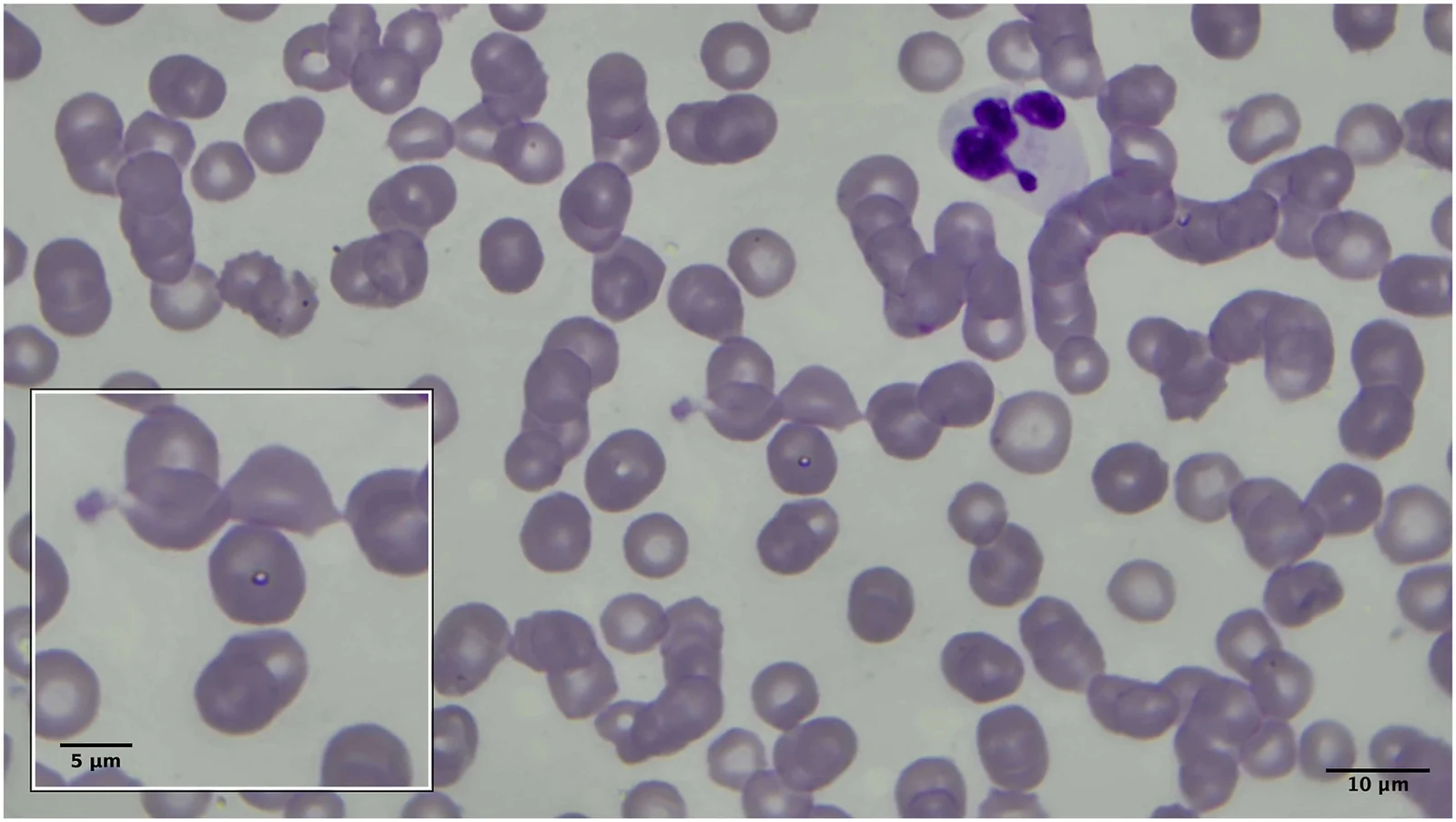
Merozoite of *Cytauxzoon* sp. in a blood smear made from peripheral blood sample of a *Conepatus semistriatus.* Panoptic stain (Laborclin, Pinhais, PR, Brazil), x1000 total magnification and inset shows a higher-magnification view.

Both samples were positive for *gapdh* in cPCR. Near full-length 18S rRNA sequences (∼1.400 bp) were obtained and deposited in GenBank (PZ654714 and PZ654715). Although near full-sequence 18S rRNA were obtained, phylogenetic analysis and genetic calculations were conducted using high-quality fragments (993 bp), corresponding to the region shared among all sequences included in this study. This approach ensured proper alignment and increased the reliability of the phylogenetic inference.

BLAST analysis of the two sequences revealed high nucleotide identity with *Cytauxzoon* spp. available in GenBank. Both sequences showed 100% query coverage and ≥ 99.3% identity with *C. brasiliensis* (PQ724420), and *C. felis* (MT904032; GU903911) indicating close genetic affinity with recognized *Cytauxzoon* especies (Table 3).

**Table 3:** BLASTn results showing nucleotide identity of the 18S rRNA sequences obtained in this study with representative *Cytauxzoon* species available in GenBank.

Sequence	Query length (bp)	Species	GenBank accession	% Identity	Query coverage	E-value
**ID 1**	993	*C. brasiliensis*	PQ724420	99.3%	100%	0.0
		*C. felis*	MT904032	99.3%	100%	0.0
		*C. felis*	GU903911	99.3%	100%	0.0
**ID 2**	993	*C. brasiliensis*	PQ724420	99.4%	100%	0.0
		*C. felis*	MT904032	99.4%	100%	0.0
		*C. felis*	GU903911	99.4%	100%	0.0

The phylogenetic tree inferred from partial 18S rRNA gene sequences (n = 155; 1056 bp) revealed the presence of three major and well-supported clades within the genus *Cytauxzoon* (Fig. 2). The first clade comprised sequences of African and Asian origin, including sequences associated with members of the families Viverridae and Herpestidae, such as *C. paradoxurus* and *Cytauxzoon* sp. from *Suricata suricatta*.

**Fig. 2:**
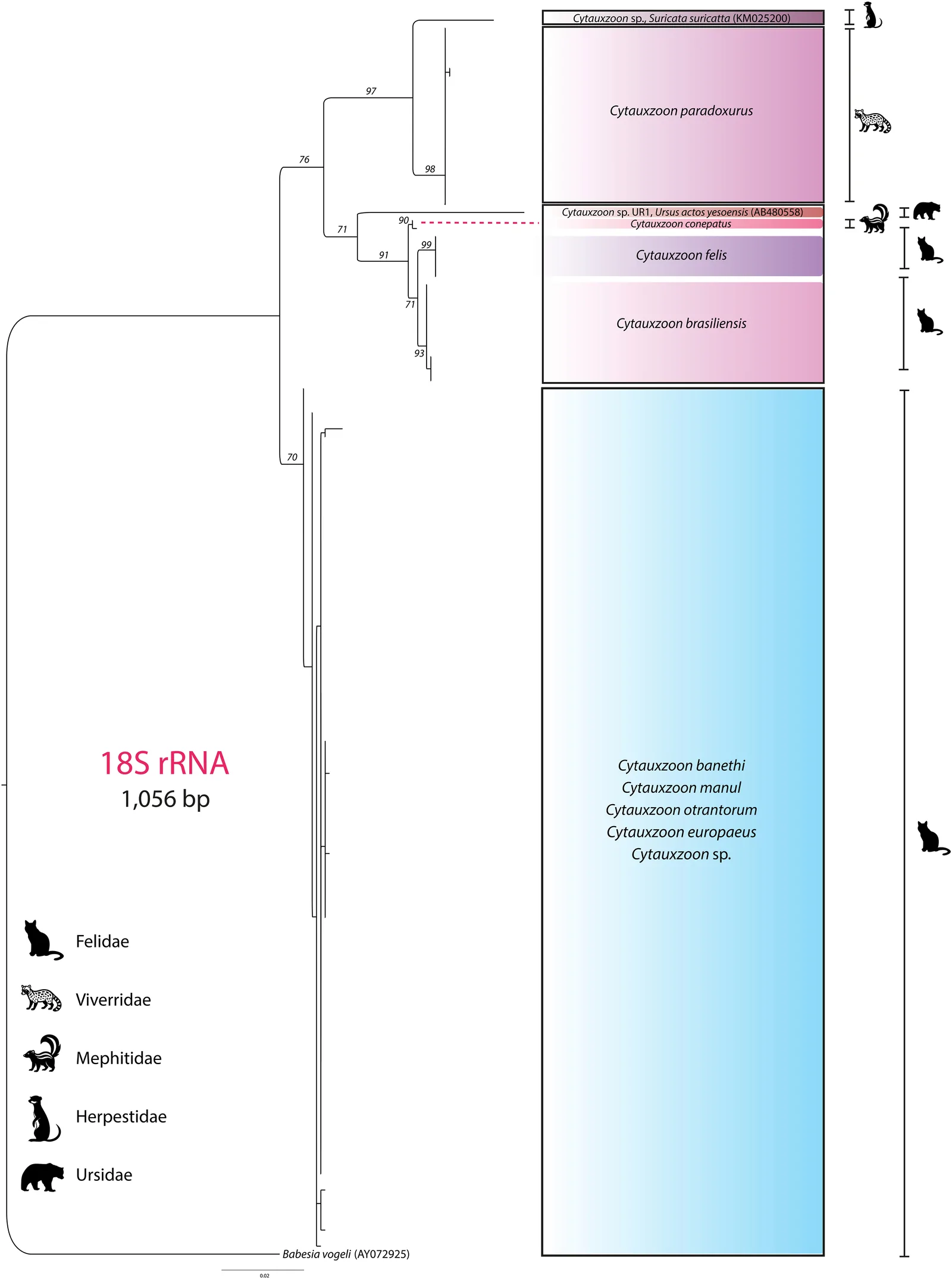
Maximum-likelihood phylogenetic tree of *Cytauxzoon* genus based on 155 sequences of the partial 18S rRNA gene (1056 bp). *Babesia vogeli* (AY072925) was used as outgroup.

The second clade encompassed sequences of American origin and included *C. felis*, *C. brasiliensis* and *Cytauxzoon* haplotypes detected in *Conepatus semistriatus*. Within this clade, the sequences obtained in the present study formed a distinct and well supported monophyletic subclade (bootstrap 90%), clearly separated from all other American *Cytauxzoon* species included in the analysis. The third clade consisted of European and Asian sequences, including *C. banethi, C. otrantorum, C. europaeus. C. manul,* and a *Cytauxzoon* sp. sequence obtained from a brown bear (*Ursus arctos yesoensis*).

Pairwise nucleotide comparisons were performed for the 18S rRNA gene to quantify the genetic divergence between the two obtained sequences and representative *Cytauxzoon* species included in the alignment. The highest nucleotide identity values were observed in comparisons with American *Cytauxzoon* species, particularly *C. brasiliensis* and *C. felis*, with similarity values ranging from 99.3 to 99.4% and corresponding p-distance of 0.006-0.007. In contrast, comparisons with European and Asian *Cytauxzoon* species, including *C. europaeus*, *C. otrantorum*, and *C. manul,* yielded lower similarity values (96.68-96.98%) and substantially higher p-distance values (0.030-0.033). The greatest divergence was observed in comparisons with *C. paradoxurus*, which showed similarity values around 96.19% and p-distance values up to 0.038.

Both samples successfully amplified the *cytb* gene by cPCR; however, only one sequence (ID 2; GenBank accession number: PZ660158) presented sufficient quality for downstream analysis. A partial *cytb* sequence of 665 bp was obtained corresponding to approximately 60% of the gene length and was used for subsequent molecular analyses.

BLASTn analysis revealed the highest nucleotide identity with *Cytauxzoon felis* (93.09%; accession number KC207821) and *Cytauxzoon brasiliensis* (92.79%; accession number PP588457), with 100% query coverage and E-values of 0.0. Additional high-scoring alignments were consistently recovered with other sequences of *Cytauxzoon felis* and *Cytauxzoon brasiliensis,* showing nucleotide identities ranging from 92.26% to 93.02%.

The phylogenetic tree inferred from the partial *cytb* gene fragment (n = 124; 622bp) revealed a structured topology, with sequences clustering into well-defined groups corresponding to previously described *Cytauxzoon* species (Fig. 3). Distinct clades were observed for *C. brasiliensis* and European and Asian species, including *C. europaeus*, *C. banethi, C. manul,* and *C. otrantorum.*

**Fig. 3:**
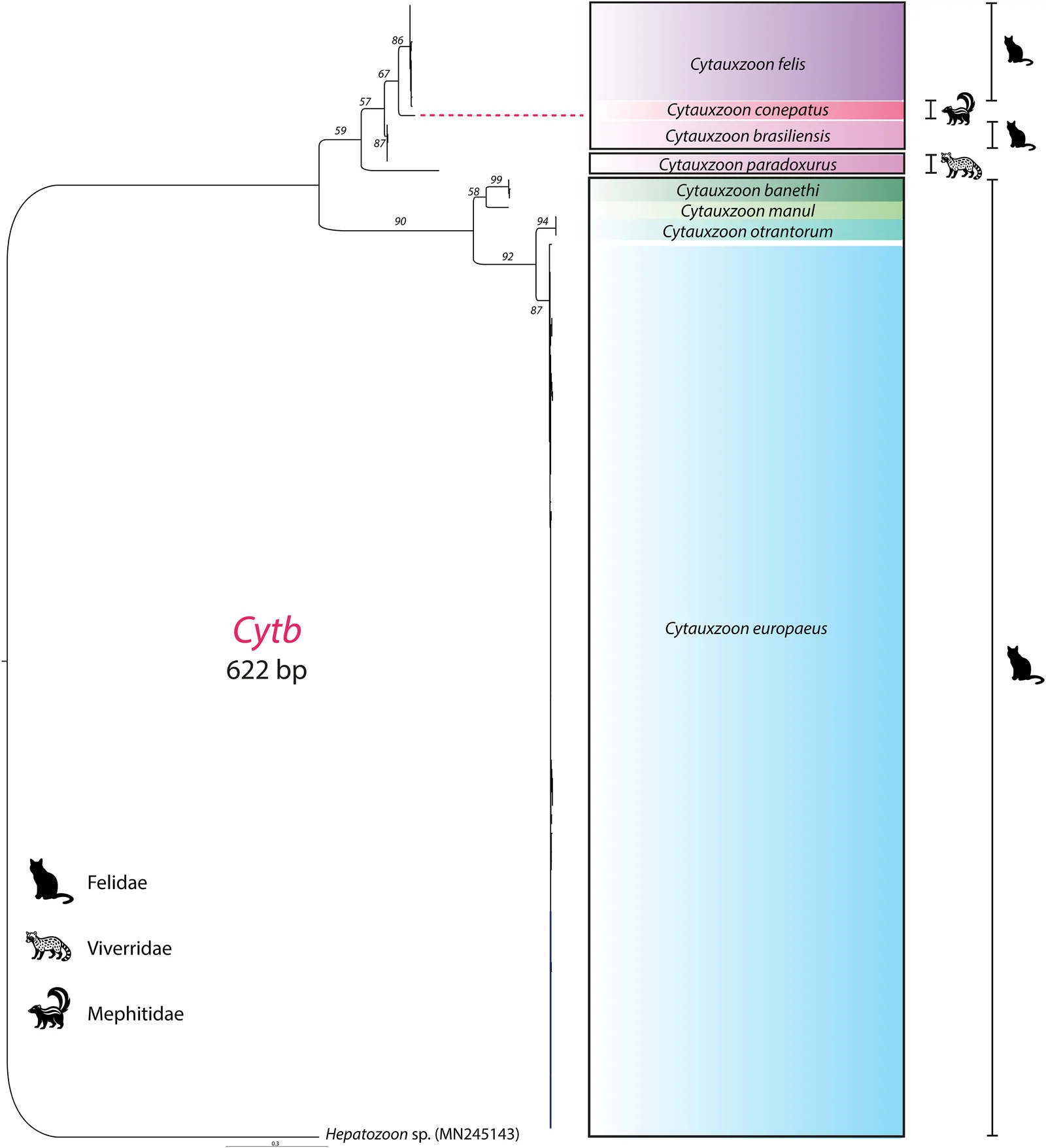
Maximum-likelihood phylogenetic tree of *Cytauxzoon* genus based on 124 sequences of the partial *cytb* gene (622 bp). *Hepatozoon* sp. (MN245143) was used as outgroup.

The sequences obtained in the present study did not cluster with any of the currently recognized species-level clades. Instead, it formed an independent branch with the phylogenetic tree, supported by a moderate bootstrap value. This topology indicates a distinct separation of the sequence from all reference *Cytauxzoon* taxa included in the analysis, despite its position within the broader assemblage of American *Cytauxzoon* sequences.

Pairwise genetic distance analysis based on the partial *cytb* gene revealed substantial variation in p-distance values between the haplotype obtained in the present study and reference *Cytauxzoon* species. Comparisons with American species yielded the lowest p-distance values, with *C. felis* showing values ranging from 0.074 to 0.077 and *C. brasiliensis* ranging from 0.082 to 0.084. In contrast, considerably higher p-distance values were observed in comparisons with European and Asian *Cytauxzoon* species. BLASTn analysis of the partial *cox-1* gene sequences obtained from the two striped hog-nosed skunks (Genbank accession number: PZ377279 and PZ377280) revealed a high degree of similarity with *Cytauxzoon* species available in GenBank. The ID 1 sequence yielded a 604 bp fragment that exhibited 100% query coverage and highest nucleotide identity with *Cytauxzoon brasiliensis* (91.49%; PQ724009), followed by *C. felis* sequences showing approximately 90.45% identity (e.g. PQ724011, MT916250). The second haplotype (ID 2), comprising a 670 bp fragment, also exhibited 100% query coverage, with the highest identity values observed for *C. brasiliensis* (91.23%; PQ724009) and *C. felis* (up to 91.32%; PQ724011 and related accessions). In both, no *cox-1* sequences from *Cytauxzoon* species showed identity values exceeding 92%, indicating a clear genetic divergence from all reference sequences currently available.

The phylogenetic tree constructed based on the partial *cox-1* gene fragments (n = 98; 552 pb) revealed a well-defined topology, showing a clear separation between the American and European/Asian *Cytauxzoon* sequences (Fig. 4). Within the American assemblage, *C. felis* and *C. brasiliensis* sequences clustered together as a distinct subclade. The sequences obtained in the present study did not clustered within either the *C. brasiliensis* or *C. felis* subclade. Instead, they formed a distinct branch, strongly supported by a bootstrap value of 95%, that diverged at a deeper node and shared a common ancestor with *C. felis* and *C. brasiliensis.*

**Fig. 4:**
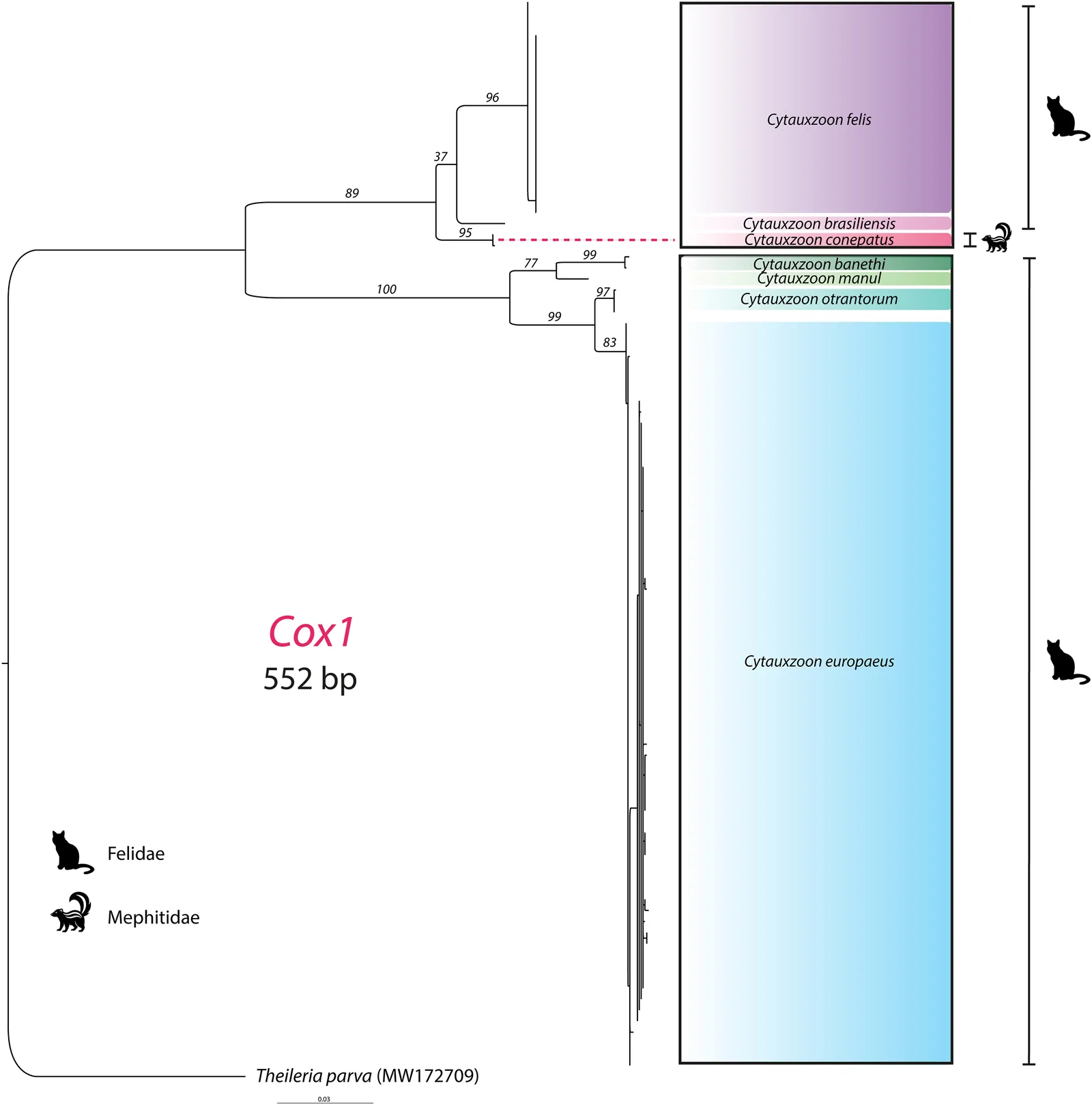
Maximum-likelihood phylogenetic tree of *Cytauxzoon* genus based on 98 sequences of the partial *cox-1* gene (552 bp). *Theileria parva* (MW172709) was used as outgroup.

In contrast, all European and Asian *Cytauxzoon* species, including *C. europaeus, C. banethi, C. manul* and *C*. *otrantorum*, clustered in a separated clade, clearly segregated from the American sequences.

The analysis of genetic distance showed consistency between the sequences in the present study and reference *Cytauxzoon* species. Comparisons between the two sequences showed very low p-distance values (<0.01). Comparisons between the haplotypes and *Cytauxzoon felis* and *C. brasiliensis* resulted in p-distance values ranging from approximately 0.087 to 0.091. Higher p-distance were observed in comparison with European and Asian *Cytauxzoon* species, which consistently presented greater genetic distances relative to the haplotypes from the present study.

In the broader 18S rRNA phylogenetic analysis, the sequences were positioned within the *Cytauxzoon* clade as a sister to the clade comprising *C. felis* and *C. brasiliensis*, with 98% bootstrap support (Supplementary File 2).

New Species Description.

Order Piroplasmida Wenyon, 1926.

Family Theileriidae du Toit, 1918.

Genus *Cytauxzoon* (Neitz and Thomas, 1948*)*

*Cytauxzoon conepatus* **n. sp** Rêgo, Duarte e Paludo.

**Type-host**: Striped hog-nosed skunk (*Conepatus semistriatus*)

**Type-locality**: Barreiras, (11.8063° S, 46.0361° W), Bahia, Brazil.

**Type-material:** the genomic DNA extracted from blood was also deposited under the PZ654714 and PZ654715 accession number for the 18S rRNA gene, PZ660158 for the *cytb* and PZ377279 and PZ377280 for the *cox-1* gene. A thin-stained blood smear from *Conepatus semistriatus* from Barreiras with ring-shaped merozoites containing halotype were deposited in the Laboratório de Patologia Clínica Veterinária, Faculdade de Agronomia e Medicina Veterinária (FAV/UnB), Universidade de Brasília, Brasília, Distrito Federal, Brazil.

**Vector**: currently unknown.

**Diagnosis**: the organism is a species of piroplasmid of the genus *Cytauxzoon,* distinct from others species of the genus in a new host based on DNA sequences and forming a monophyletic clade.

**Zoobank registration**: In accordance with Article 8.5 of the Internacional Code of Zoological Nomenclature (ICZN), this work and the nomenclature act proposed herein have been registered in Zoobank. The Life Science Identifier (LSID) for this publication is rn:lsid: zoobank.org:pub:142200B5-EAE5-4FAF-B4B7-325E02D22085. The LSID for the new name *Cytauxzoon conepatus* **n. sp.** is urn:lsid:zoobank.org:act98039AE0-5DD1-44BA-A5D0-E408321DC1A4.

**Etymology:** The new species is named *conepatus* in reference to *Conepatus semistriatus*, the striped hog-nosed skunk from which the type of material was obtained.

## 4 Discussion

This study presents the first record of infection by *Cytauxzoon* species in *Conepatus semistriatus*, a member of the family Mephitidae, expanding current knowledge about the diversity of hosts associated with this hemoprotozoan. The present finding constitutes the fourth report of *Cytauxzoon* infection in non-felid hosts, adding to the records previously described in meerkats (*Suricata suricatta*) (Leclaire et al., 2015) in southern Africa, in brown bears and Asian bears (*Ursus arctos yesoensis* and *Ursus thibetanus japonicus*) (Moustafa et al., 2020) in Japan, civets (*Paradoxurus musangus*) in Singapore (Chong et al., 2025), and hyenas in Iran (*Hyaena hyaena*) (Sazmand et al., 2026).

The records of natural infection by *Cytauxzoon* were predominantly concentrated in wild and domestic felids from America, Europe and Asia. A similar scenario is found in South America, where molecular studies on *Cytauxzoon* sp. are carried out in wild felids under in situ and ex-situ conditions, revealing the occurrence of infection in hosts such as the little spotted cat (*L. tigrinus*) (Duarte et al., 2024), ocelot (*L. pardalis*), puma (*Puma concolor*) (André et al., 2009; Bittencourt et al., 2025), Lions (*Panthera leo*) (Peixoto et al., 2007), jaguar (*Panthera onca*) (Calchi et al., 2025a; Fagundes-Moreira et al., 2022), just like in domestic cats (André et al., 2009; Carvalho et al., 2024; de Oliveira et al., 2022; Palmer et al., 2022; Raimundo et al., 2021).

In the United States, *C. felis* infection in domestic cats is often severe and potentially fatal, causing systemic signs such as lethargy, fever, dyspnea, hemolytic crises, and jaundice, with common laboratory abnormalities including leukopenia, anemia, thrombocytopenia, and hyperbilirubinemia (Kastl and Springer, 2023; Reichard et al., 2021). In contrast, *Cytauxzoon* infections in Brazilian domestic cats have generally been subclinical, with no major clinical or laboratory abnormalities or reported deaths (André et al., 2022; Carvalho et al., 2024; de Oliveira et al., 2022; Raimundo et al., 2021). However, a recent study of *C. brasiliensis*-positive cats reported mild anemia, inflammatory leukogram, and thrombocytopenia, although coinfections with other haemopathogens were not assessed (Brandão et al., 2026).

In Brazil, knowledge about the clinicopathological changes in *Cytauxzoon* infections in wild animals is limited, given that few studies combine clinicopathological and molecular data, which may represent a loss of important data on the pathophysiology of local *Cytauxzoon* strains, such as *C. brasiliensis*. De Paula et al. (2022) report the presence of anemia, cachexia and dehydration in *Puma concolor*, which the authors believe are not related to the *Cytauxzoon* infection, but rather to the clinical picture of burns presented by the patient.

The specimens sampled in this study were clinically healthy, and the absence of specific hematological and biochemical reference values for *C. semistriatus* makes it difficult to accurately interpret findings related to this *Cytauxzoon* infection. Using hematological and biochemical reference values for *Mephitis mephitis*, another representative of the Mephitidae family, the results suggest that the evaluated parameters are within normal limits, and in association with the good clinical condition of the sampled individuals, suggests that the identified parasite exhibits the same behavior as those reported in domestic cats and wild felids in Brazil, resulting in a subclinical infection. Despite this, in Brazil there are already isolated reports of the fatal form of cytauxzoonosis in lions (*Panthera leo*) (Peixoto et al., 2007) and jaguar (*Panthera onca*) (Guizelini et al., 2021). This corroborates the observation made by André et al. (2022), suggesting that different *Cytauxzoon* genotypes may occur in Brazil and exhibit varying degrees of pathogenicity and host susceptibility.

In Europe, the diversity of species in this genus has increased in recent decades with the description of new species such as *C. europaeus, C. banethi,* and *C. otrantorum*, identified in wild cats, including European wildcat (*Felis silvestris*) and the Eurasian lynx (*Lynx lynx*) (Obiegala et al., 2024; Panait et al., 2021). *C. banethi* has also recently been detected in striped hyena (*Hyaena hyaena*) (Sazmand et al., 2026). Similarly, knowledge about new hosts has increased for the Asian species, with the more recently described species of the genus, *Cytauxzoon paradoxurus,* in civets (*Paradoxurus musangus*) (Chong et al., 2025) and the description of the occurrence of *C. manul* in eurasian lynx (*Lynx lynx*) in northwestern China (Cui et al., 2024).

Infections caused by *Cytauxzoon* recorded in Brazil were, for a long time, documented based on short 18S rRNA fragments and showed high similarity to *C. felis*. However given the limited resolution of the marker and the limited sequence length, the species-level identity of these haplotypes remains uncertain (de Paula et al., 2022; Guizelini et al., 2021). The description of *C. brasiliensis*, a native South American species, by Duarte et al. (2024) marked a milestone in understanding the diversity of the genus in South America, as it was initially associated with the little spotted cat (*Leopardus tigrinus*). More recently, others host for *C. brasiliensis* have been reported in other felid hosts, including ocelots (*Leopardus pardalis*) (Calchi et al., 2025a), cougars (*Puma concolor*) (Bittencourt et al., 2025) and domestic cats (Brandão et al., 2026), demonstrating that the parasite has a broader host range than previously recognized. This finding underscores the need for further ecological and molecular studies to elucidate the range of niches occupied by this newly identified species.

The detection of *Cytauxzoon conepatus* n. sp. in *Conepatus semistriatus* in the present study opens important hypotheses about the breadth of the spectrum of hosts that can be infected by *Cytauxzoon* species. The historical association of the parasite with species from the Felidae family may have contributed to a sampling bias that neglects other groups of carnivores, potentially masking a broader parasitological diversity. In this context, the previous absence of records of this species in studies carried out in the region should not be interpreted as evidence of rarity or restricted distribution, but possibly as a reflection of the low representation of non-felid hosts in molecular surveys.

Accordingly, *C. semistriatus* may represent a natural host for *Cytauxzoon conepatus* n. sp. since none of the animals showed clinical-laboratory changes. This pattern is consistent with hypotheses of evolutionary co-association between parasites and hosts and reinforces the importance of including non-felid carnivores in future studies on the diversity and evolution of the genus *Cytauxzoon*. A similar fact was found by Chong et al. (2025) when describing for the first time *Cytauxzoon paradoxurus*, which associates the species with civets in Southeast Asia as the natural host of the species in question.

Initial BLASTn analyses of the 18S rRNA gene sequences obtained in this study revealed a high nucleotide identity (99.3-99.4%) with both *C. brasiliensis* and *C. felis.* These similarity values are consistent with previous observations highlighting the conserved nature of the 18S rRNA gene within the genus *Cytauxzoon*, as reported by Panait et al. (2021) when describing three new European species, by Duarte et al. (2024) in the description of *C*. *brasiliensis*, and by Calchi et al. (2025) in their study on wild felines from Brazil and Argentina.

Despite the high similarity observed in BLAST analyses, phylogenetic reconstruction based on the 18S rRNA gene (n = 155; 1072 bp) revealed a clear and well-structured topology within the genus. Three majors, strongly supported clades were recovered, corresponding broadly to African-Asian, American, and Eurasian groupings.

This geographic structuring is congruent with previous large-scale phylogenetic reconstructions and reinforces the hypothesis that *Cytauxzoon* evolution is strongly influenced by biogeographic history and host association. Within the American clade, the sequences obtained in the present study formed a distinct and strongly supported monophyletic subclade (98% bootstrap), clearly separated from *C. felis* and *C. brasiliensis*.

Genetic distance analysis of the two 18S rRNA sequences of *Cytauxzoon conepatus* n. sp. revealed low p-distance values relative to American *Cytauxzoon* species (0.006–0.007), particularly *C. felis* and *C. brasiliensis*, whereas substantially greater divergence was observed in comparisons with European and Asian species (p-distance ≥ 0.030). These findings support the placement of the sequences within the American lineage of the genus. This pattern is consistent with the highly conserved nature of the 18S rRNA gene and its limited ability to discriminate among *Cytauxzoon* species, particularly closely related taxa.

In contrast, the description of *Cytauxzoon paradoxurus* by Chong et al. (2025) was based on a high p-distance for this marker, indicating an older evolutionary divergence, which explains the greater 18S rRNA resolution capacity in that specific context. Thus, the low distances observed between Neotropical *Cytauxzoon* species suggest a more recent diversification process, reinforcing the need to use more variable mitochondrial markers for adequate taxonomic delimitation of this group.

Mitochondrial markers provided additional and independent support for the phylogenetic distinction identified in this study. In the BLASTn analysis of the *cytb* gene of ID 2 (662 bp), it showed greater similarity with *Cytauxzoon felis* (93.09%) from a domestic cat in the United States (KU207821) and 92.79% with *C. brasiliensis* previously identified in *L. tigrinus* (PP588457). This value is lower than that observed by Duarte et al. (2024) between *C. brasiliensis* and *C. felis* (93.2-93.3%) and compatible with interspecific differentiation within the genus *Cytauxzoon.*

The phylogenetic tree based on the *cytb* gene revealed a structured topology, with well-defined clades corresponding to previously described species. The analyzed sequence did not group with *C. felis* or *C. brasiliensis*, forming, instead, an independent branch supported by a moderate bootstrap value.

The p-distance values observed for the *cytb* gene were considerably higher than those recorded for the 18S rRNA gene, ranging from 0.074 to 0.077 compared to *C. felis* and from 0.082 to 0.084 compared to *C. brasiliensis*. These interspecific divergence values are higher than those described by Duarte et al. (2024) between *C. felis* and *C. brasiliensis* (0.067-0.068). These interspecific divergence values, higher than those previously described between *C. felis* and *C. brasiliensis*, indicate that the analyzed sequence presents a level of genetic differentiation compatible with separation at a specific level, reinforcing its condition as an evolutionarily distinct species within the genus *Cytauxzoon*.

Analyses based on the *cox-1* gene reinforced this pattern. Both samples exhibited nucleotide identity values lower than 92% when compared with all available reference sequences, including *C. felis* and *C. brasiliensis*. In the corresponding phylogenetic tree, the sequences formed a distinct and strongly supported branch (95% bootstrap) within the American cluster, diverging from a common ancestor shared with *C. felis* and *C. brasiliensis*. The congruent signal obtained from nuclear and mitochondrial markers provide strong evidence that these Cytauxzoon sequences do not represent mere genovariants of previously described species, but rather an evolutionarily independent species consistent with differentiation at the species level.

In the broader phylogenetic analysis of Piroplasmida, *Cytauxzoon conepatus* n. sp. Clustered with the other recognized *Cytauxzoon* species, supporting its placement within the *Cytauxzoon* clade. This topology is compatible with the updated phylogenetic framework proposed by Calchi et al. (2025).

By integrating analyzes of nuclear (18S rRNA) and mitochondrial (*cytb* and *cox-1*) gene sequences, this study contributes to closing existing knowledge gaps and advances the understanding of *Cytauxzoon* diversity across the Americas. The identification of *Cytauxzoon conepatus* n. sp. exclusively in *Conepatus semistriatus* also offers important evolutionary and ecological insights.

From an ecological perspective, the recognition of new *Cytauxzoon* species in previously unsampled hosts also raises fundamental questions regarding their transmission dynamics. In North America experimental studies have proven that the ticks *Amblyomma americanum* and *Dermatocentor variabilis* are the vectors for *C. felis* (Blouin et al., 1987)*,* in South America, especially in Brazil, the *Amblyomma sculptum* is suggested as a likely vector, but there is still no experimental study to prove this hypothesis (Fagundes-Moreira et al., 2022; Santana et al., 2022). Likewise, for the species described herein, the arthropod vector involved in transmission remains unknown, underscoring the need for future studies integrating molecular, ecological and experimental approaches.

Some limitations of the present study should be acknowledged. The number of samples analyzed was limited, and only a single *cytb* gene sequence was obtained. This constraint is partly attributable to the inherent difficulties associated with capturing free-ranging wildlife, particularly *Conepatus semistriatus*, a species for which ecological and parasitological data remain limited, especially regarding host-parasite interactions in the Cerrado biome.

Additionally, the vector and other stages of the parasite's life cycle remain unknown. Nonetheless, similar limitations have been reported in several recent descriptions of new *Cytauxzoon* species. Importantly, the consistent recovery of a distinct monophyletic species across multiple genetic markers helps to mitigate these limitations. The combined use of nuclear and mitochondrial data enhances the robustness of taxonomic inferences and minimizes the risk of misinterpretations that could arise from single-locus analyses.

In summary, the molecular, phylogenetic, and genetic distance analyses conducted in this study support the recognition of *Cytauxzoon conepatus* n. sp. as a new species infecting a novel host, *Conepatus semistriatus,* in Brazil. This finding strengthens the evidence that the diversity of *Cytauxzoon* across the Americas has been underestimated and underscores the importance of multilocus approaches for accurately delimitating species within the genus. Future studies incorporating broader sampling, additional genetic markers, and ecological data will be essential to advance our understanding of the evolutionary relationships, host range, and epidemiological significance of *Cytauxzoon* species in South America.

## 5 Conclusion

This study provides the first report of infection by *Cytauxzoon conepatus* n. sp. in *Conepatus semistriatus*, thereby substantially expanding the known host range of the genus *Cytauxzoon*. Multilocus analyses based on the 18S rRNA, *cox-1,* and *cytb* genes, combined with the recovery of well-supported monophyletic clades and the absence of clinical and laboratory changes in the sampled individuals, support the recognition of an evolutionarily distinct species, likely endemic to the Brazilian Cerrado.

These findings strengthen the hypothesis that the diversity of *Cytauxzoon* species in South America has been considerably underestimated, largely due to the traditional focus on felid hosts. Expanding molecular and ecological investigations, including vector identification and broader sampling across different biomes and carnivores groups, will be crucial to clarify the true diversity, evolutionary history, and epidemiological significance of *Cytauxzoon* species on the South American continent.

## CRediT authorship contribution statement

**George Magno Sousa do Rêgo:** Writing – original draft, Methodology, Investigation, Formal analysis, Data curation, Conceptualization. **Ana Paula Nunes de Quadros:** Writing – review & editing, Methodology, Data curation, Conceptualization. **Matheus Almeida Duarte:** Writing – review & editing, Visualization, Methodology, Investigation, Formal analysis, Data curation, Conceptualization. **Liliane Maria do Rosario Batista:** Writing – review & editing, Visualization, Methodology, Investigation, Formal analysis, Data curation. **Paula Damasceno Gomes:** Writing – review & editing, Methodology, Investigation, Formal analysis, Conceptualization. **Giane Regina Paludo:** Writing – review & editing, Supervision, Methodology, Funding acquisition, Formal analysis, Conceptualization.

## Data availability

The nucleotide sequences generated in this work have been deposited in the GenBank database under accession numbers PZ654714 and PZ654715 (18S rRNA), PZ660158 (*cytb*), and PZ377279 and PZ377280 (*cox-1*). All other data supporting the findings of this study are included within the article and its supplementary material.

## Funding

This work was supported by the Coordination for the Improvement of Higher Education Personnel (CAPES), Brazil, through the provision of a doctoral scholarship to the author. Partial financial support was also provided by the Research Support Foundation of the Federal District (FAPDF), Brazil, which enabled the acquisition of laboratory supplies, sequencing analyses, and the purchase of equipment. This support was granted under FAPDF Call No. 09/2022 (SEI Process No. 0019300002290/2022-93).

## Declaration of competing interest

The authors declare that they have no known competing financial interests or personal relationships that could have appeared to influence the work reported in this paper.
