# Supplementary material for: Cytauxzoon conepatus n. sp. (Piroplasmida: Theileriidae) infecting Conepatus semistriatus (Mephitidae) in Brazil
Source: Int J Parasitol Parasites Wildl. 2026 Oct 3;31:101297. doi: 10.1016/j.ijppaw.2026.101297 (PMC13671328; doi:10.1016/j.ijppaw.2026.101297)
Supplement: Supplementary file 1 [file mmc2.pdf]

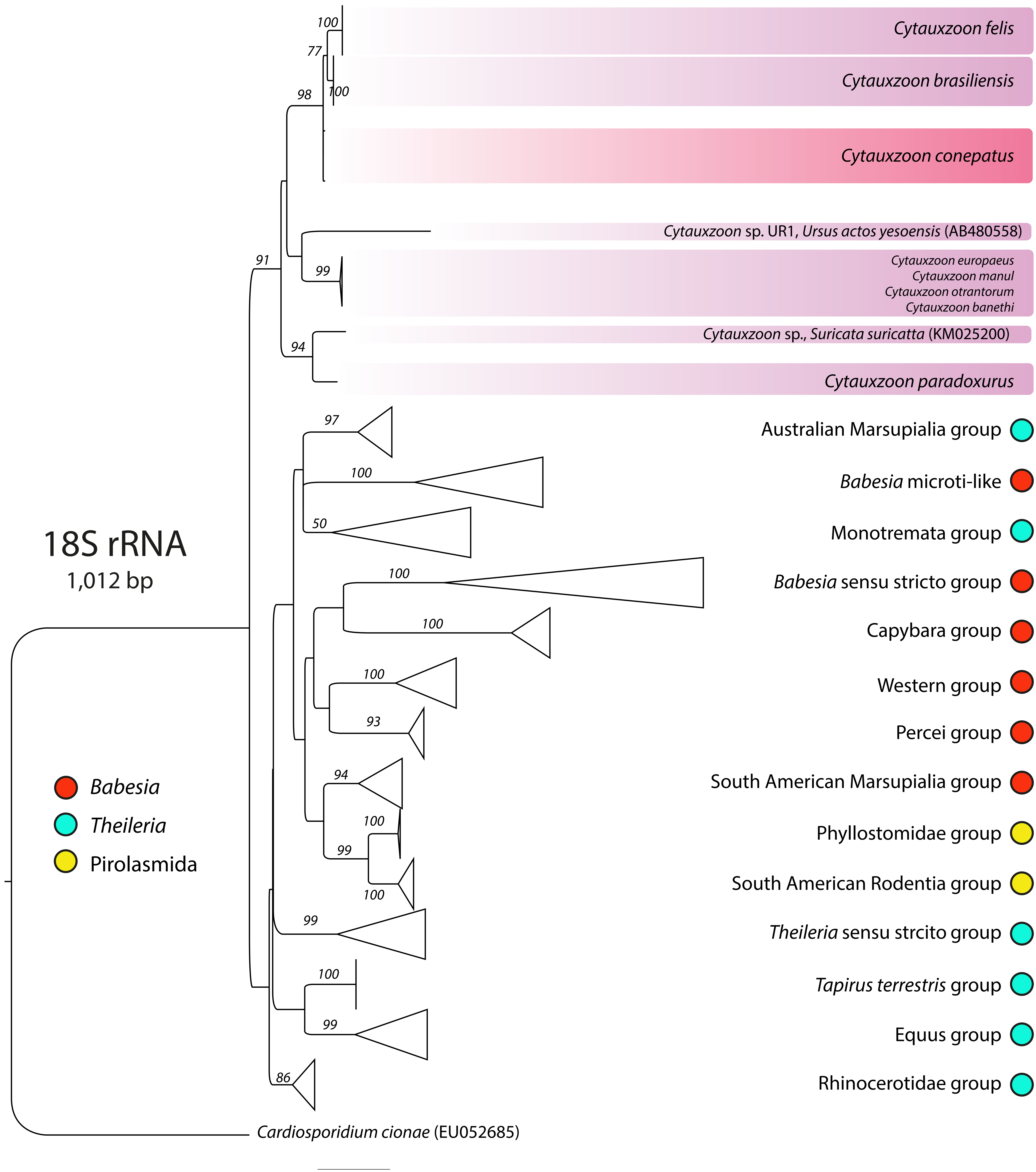

Cytauxzoon felis
Cytauxzoon brasiliensis
Cytauxzoon conepatus
Cytauxzoon sp. UR1, Ursus actos yesoensis (AB480558)
Cytauxzoon europaeus
Cytauxzoon manul
Cytauxzoon otrantorum
Cytauxzoon banethi
Cytauxzoon sp., Suricata suricatta (KM025200)
Cytauxzoon paradoxurus
Australian Marsupialia group
Babesia microti-like
Monotremata group
Babesia sensu stricto group
Capybara group
Western group
Percei group
South American Marsupialia group
Phyllostomidae group
South American Rodentia group
Theileria sensu strcito group
Tapirus terrestris group
Equus group
Rhinocerotidae group
18S rRNA
1,012 bp
Babesia
Theileria
Pirolasmida
Cardiosporidium cionae (EU052685)
0.05
